# From Theory to Practice: Bone Health in Women with Early Breast Cancer Treated with Aromatase Inhibitors

**DOI:** 10.3390/curroncol28020104

**Published:** 2021-02-26

**Authors:** Leonor Vasconcelos de Matos, Leonor Fernandes, Maria Teresa Neves, Fátima Alves, Mafalda Baleiras, André Ferreira, Pedro Giesteira Cotovio, Tiago Dias Domingues, Mariana Malheiro, Ana Plácido, Maria Helena Miranda, Ana Martins

**Affiliations:** 1 Department of Medical Oncology, Hospital de São Francisco Xavier, Centro Hospitalar de Lisboa Ocidental, 1449-005 Lisbon, Portugal; fernandes.leonor@gmail.com (L.F.); mtneves@chlo.min-saude.pt (M.T.N.); fnalves@chlo.min-saude.pt (F.A.); abaleiras@chlo.min-saude.pt (M.B.); fafferreira@chlo.min-saude.pt (A.F.); mmrodrigues@chlo.min-saude.pt (M.M.); applacido@chlo.min-saude.pt (A.P.); mhmiranda@chlo.min-saude.pt (M.H.M.); ammourao@chlo.min-saude.pt (A.M.); 2 CEAUL, Faculdade de Ciências, Universidade de Lisboa, 1749-016 Lisbon, Portugal; pepedro97@gmail.com (P.G.C.); tmdomingues@fc.ul.pt (T.D.D.); 3 Department of Medical Oncology, Hospital Cuf Tejo, 1350-353 Lisbon, Portugal

**Keywords:** breast cancer, aromatase inhibitors, osteoporosis, fracture

## Abstract

Aromatase inhibitors (AI) are extensively used as adjuvant endocrine therapy in post-menopausal women with hormone receptor-positive early breast cancer (HR+ EBC), but their impact on bone health is not negligible. This work aimed to assess bone loss, fracture incidence, and risk factors associated with these events, as well as the prognostic influence of fractures. We have conducted a retrospective cohort study of women with HR+ EBC under adjuvant therapy with AI, during a 3-year period. Four-hundred-and-fifty-one eligible women were reviewed (median age 68 years). Median time under AI was 40 months. A fracture event occurred in 8.4%, mostly in the radium and femoral neck and in older women (mean 74 vs. 68 years, *p* = 0.006). Age (OR 1.01, 95% CI 1.01–1.07, *p* = 0.024) and time under AI (OR 1.02, 95% CI 1.00–1.04, *p* = 0.037) were independent predictors of fracture, with a fair discrimination (AUC 0.71). Analysis of disease-free survival according to fracture event varied between groups, disfavoring the fracture cohort (at 73 months, survival 78.6%, 95% CI, 47.6–92.4 vs. 95.6%, 95% CI, 91.2–97.8, *p* = 0.027). The multivariate model confirmed the prognostic impact of fracture occurrence (adjusted HR of 3.17, 95% CI 1.10–9.11; *p* = 0.032). Bone health is often forgotten, despite its great impact in survivorship. Our results validate the pathophysiologic link between EBC and bone metabolism, which translates into EBC recurrence. Further research in this area may help refine these findings. Moreover, early identification of women at higher risk for fractures is warranted.

## 1. Introduction

### 1.1. Treatment-Induced Bone Loss (TIBL) in Early Breast Cancer

In early breast cancer (EBC), endocrine therapy (ET) is recommended for all women with detectable expression of estrogen receptors. The choice of ET is primarily defined by the menopausal status, also weighting possible side effects and contraindications [1]. In post-menopausal women, aromatase inhibitors (AI) are commonly the adjuvant therapy of choice as they have shown modest but significant increased efficacy and survival when compared with tamoxifen, in large phase III randomized trials and subsequent meta-analysis [2]. AI act by decreasing serum levels of circulating estrogens, blocking the conversion of androgens to estrogen in peripheral tissues, through inhibition of the enzyme aromatase [3]. As the bone is an endocrine-responsive organ, the decrease in circulating estrogens accelerates bone resorption and thus AI represent a major issue for bone health.

Additionally, other systemic therapies for breast cancer can contribute to the deregulation of bone turnover, either through effects on gonadal steroid hormone production or by inhibiting peripheral aromatization into estrogen, therefore raising the probability of osteopenia, osteoporosis, and bone fractures. These include chemotherapy, radiation therapy, and medications such as glucocorticoids. Chemotherapy, in particular, is not only an independent risk factor for bone mineral density (BMD) loss, but also leads to gonadal dysfunction inducing premature ovarian failure in premenopausal women [4,5,6]. Radiation toxicities include not only BMD loss and increased risk of fractures, but also avascular necrosis, medullary fibrosis, and secondary malignancies [7]. The status of induced menopause, either due to cytotoxic chemotherapy, gonadotropin releasing-hormone (GnRh) agonists, or oophorectomy, can also be, by itself, a risk factor for loss of bone mass [8].

### 1.2. Recommendations for the Prevention of TIBL

For women receiving antiestrogen therapy for EBC, guidelines have been published aiming for a systematic and standardized assessment and approach to bone health in this population [9,10,11,12,13]. Briefly, recommendations state that in all women with EBC proposed for treatment with AI, a structured plan targeting bone health should be carried out, with the following required steps: at baseline, evaluation of risk factors for BMD loss and fractures (including: older age, smoking habits, corticosteroid therapy, body mass index lower than 20 kg/m^2^; personal history of fragility fracture), measuring of BMD (T-score, Z-score) together with advice regarding health lifestyles, as strength exercises, smoke, and alcohol cessation; adequate supplements are also demanded: calcium (1000 mg/day) and vitamin D (1000–2000 U/day). In selected cases, bone targeted agents should also be started in order to improve or prevent further BMD loss. These cases include, according to European Guidelines, patients with a T-score < −2.0, or any 2 of the following risk factors: aged 65 years or older, T-score < −1.5, Smoking habits, BMI < 24; Family history of hip fracture, Personal history of fragility fracture above age 50, or oral glucocorticoid use for >6 months [10].

Antiresorptive agents, especially zoledronic acid, have shown benefit in preventing BMD loss when administered every 6 months for a 3-year period. Therefore, they are recommended for all BC women taking adjuvant AI when osteoporosis or other risk factors are present.

Despite these recommendations, translation to clinical practice has been slow and timorous, mostly regarding side effects of bisphosphonates that, although important are rarely severe and can be prevented (for example, osteonecrosis of the jaw and hypocalcemia). Additionally, with some exceptions [14,15,16], the population included in several of these studies, after selection and exclusion criteria, rarely reflects the real-world population seen in routine clinical practice [17,18,19,20].

Besides impairing significantly quality of life, bone fractures contribute to morbidity, mortality, and higher health-associated costs [9,21] and survivors of BC appear to have a 5–10% increased risk of fractures [22,23]. Furthermore, in postmenopausal women taking AI, BMD loss is 2 to 4 times higher than in those not taking AI [22]. AI therapy is usually undertaken from 2 to 5 years and some trials of 5-year AI therapy have described an increase in absolute fracture risk close to 10%, indicating that 1 in every 10 women with BC taking AI will suffer a fracture [20,23].

This work aimed primarily to assess real-world BMD loss associated with the use of AI in EBC women and its impact on fracture incidence. Implementation of preventive measures for bone loss was also studied. Additionally, we have exploratorily analyzed the impact of a fracture event on disease-free survival. This study thus intended to analyze the real-world evidence on the importance of assessing bone health in women with BC taking AI, therefore contributing for effective translation of guidelines into practice.

## 2. Materials and Methods

We have conducted an observational, descriptive, retrospective cohort study, performed in the Oncology Department of a tertiary university hospital. All women under follow-up after EBC in our Department, between 1/01/2015 and 1/01/2018, were included. Patients were amenable for enrollment if they were aged 18 years or older, had a histologically confirmed diagnosis of BC and were receiving treatment in the adjuvant setting with aromatase inhibitors. A minimum of 3 months under AI and 3 years of follow-up after AI start were necessary for inclusion. Women were excluded if there was lack of recorded medical data or they had clinical or radiological evidence of metastatic disease.

Patients were followed until disease recurrence, death, or censored if alive at end of follow-up date (February 2019). Codification of fracture events was assessed according to the International Classification of Diseases (ICD), 10th revision. Demographic and clinical data were extracted from medical files, and variables included were age, breast cancer biological subtypes, current glucocorticoid therapy (defined as at least three months under treatment with glucocorticoids, on a dose >30 mg of prednisolone or equivalent per month), smoking habits (defined as current smoking of at least 1 pack per month), and alcohol consumption (defined as > 1 drink per day). Information regarding medication was obtained from the hospital pharmacy recordings. BMD and T-score information were obtained from bone osteodensitometry exams, performed by the institution. First bone densitometry was performed in the time period that went from 3 months before AI start to 6 months after AI start. Subsequent osteondensitometry exams were requested according to physician’s judgement.

Primary outcome was fracture incidence, defined as any new bone fracture event. The secondary endpoint was disease-free survival, defined as the number of months until disease relapse, death from any cause, or censored if alive and with no relapse at follow-up date.

Analysis of normality was undertaken using the Shapiro–Wilk test. To compare the study groups, the Wilcoxon–Mann–Whitney test was used for continuous variables and the χ2 test of independence for categorical variables. Fisher’s exact test was used when expected frequency was <5. Pearson’s correlation was used for weight assessments. Logistic regression models were performed to study the effect of explanatory variables on outcome variable, with a stepwise approach for multivariate analysis. For each factor, we have calculated the adjusted odds ratio (OR) and 95% Confidence interval (CI) using maximum likelihood estimation. The predictive performance of the model was evaluated using ROC curve. Disease-free survival plots were built using Kaplan–Meier methods, according to occurrence of fracture events. Between-group differences between survival rates were tested for significance using the log-rank test. Cox proportional hazards models (estimated hazard ratios and 95% CIs) were used to study the association between potential prognostic factors and survival.

All analysis were performed using R software version 3.5.2., R-studio version 1.1.456 and Stata 15.1 software (StataCorp LLC). All results with a *p*-value less than 0.05 were considered statistically significant.

## 3. Results

### 3.1. Clinical Characteristics of the Study Population

This study included 451 women with EBC who underwent at least 3 months of therapy with AI. Median age was 68 years (30–98, min–max) and 84% (*n* = 381) were spontaneously in menopause, whereas 16% (*n* = 70) were on menopause due to ovarian function suppression. Most patients underwent curative treatment with chemotherapy (64%) and radiotherapy (79%) and median time under adjuvant AI of the evaluated population was 40 months (3–114). Most patients had an abnormal BMD on first bone densitometry (24% with osteoporosis and 51% with osteopenia). Among additional risk factors for BMD loss and subsequent fractures, 12% women had smoking habits, 4% alcohol consumption, 4% glucocorticoid use, and 7% had history of previous fracture. Despite risk factors, only 16% patients were under antiresorptive therapy. Table 1 summarizes clinical and demographic characteristics of the study population. 

### 3.2. Fracture Incidence

Of the 451 EBC women under AI therapy, 8.4% (*n* = 38) had a fracture event. In the fracture group, median time from AI start to fracture occurrence was 49 months (3–110). All but 3 of these women were spontaneously in menopause. Only 67% (*n* = 24) were taking Calcium and Vitamin D supplementation (versus 69% in the non-fracture group, *p* = 0.86) and 22% (*n* = 8) (versus 15% in the non-fracture group) were prescribed therapy with bisphosphonates.

Fracture of the radium was the most common, happening in 31% cases (*n* = 12), followed by femoral neck fractures (19%, *n* = 7). Most commonly, fracture diagnosis occurred after a fall (66%, *n* = 25). In most cases, fracture treatment was conservative, with 37% cases (*n* = 14) needing surgical approach. Important to state that no fracture event was related to underlying bone metastases.

When comparing women who suffered a fracture event with women who did not, age was significantly higher in the fracture group (mean 74 vs. 68, *p* = 0.006). 

### 3.3. Bone Mineral Density Loss

First osteodensitometry after starting AI therapy was performed, in median, at 8 months (2–20), but 31% (*n* = 40) women did not undergo a bone densitometry during AI treatment. Only 7% (*n* = 30) had a BMD assessment performed before starting adjuvant AI. The exam was thereafter undertaken every 1 to 2 years until the end of AI therapy. Subsequent BMD loss was observed in 50% patients (*n* = 224), either classified as osteopenia or osteoporosis.

**Figure 1:**
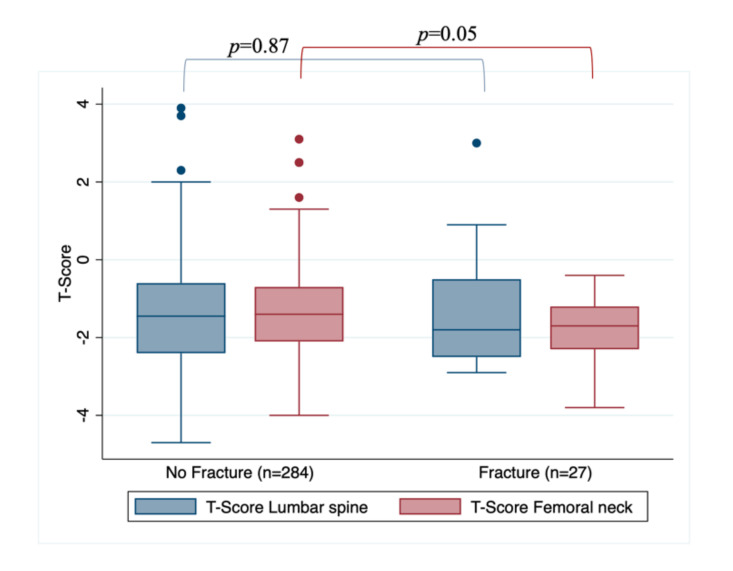
Association between lumbar and femoral T-scores and fracture occurrence.

When assessing BMD loss, according to T-score in femoral neck and lumbar spine, the overall cohort showed a median T-score in Lumbar spine of −1.5 (range: −4.7 to 3.9) and −1.5 (range: −4 to 3.1) in femoral neck. The fracture group had a median T-score in Lumbar spine of −1.8 (range: −2.9 to 3) and −1.7 (range: −3.8 to −0.4) in femoral neck. In women without fracture occurrence, median T-score in lumbar spine was −1.45 (range: −4.7 to 3.9) and −1.4 (range: −4 to 3.1) in femoral neck, this last differing from the fracture group (*p* = 0.05). Figure 1 shows the association between lumbar and femoral T-scores and fracture incidence, demonstrating the difference in T-scores between the fracture and non-fracture cohorts.

### 3.4. Risk Factors for Bone Mineral Density Loss and Fracture Events 

Accounting for known risk factors for BMD loss and after univariate analysis, we fit a multivariate regression model to assess independent predictors of bone fracture events. As shown in Table 2, age (OR 1.01, 95% CI 1.01–1.07, *p* = 0.024) and time under AI (OR 1.02, 95% CI 1.00–1.04, *p* = 0.037) were independent predictors of fracture events.

**Figure 2:**
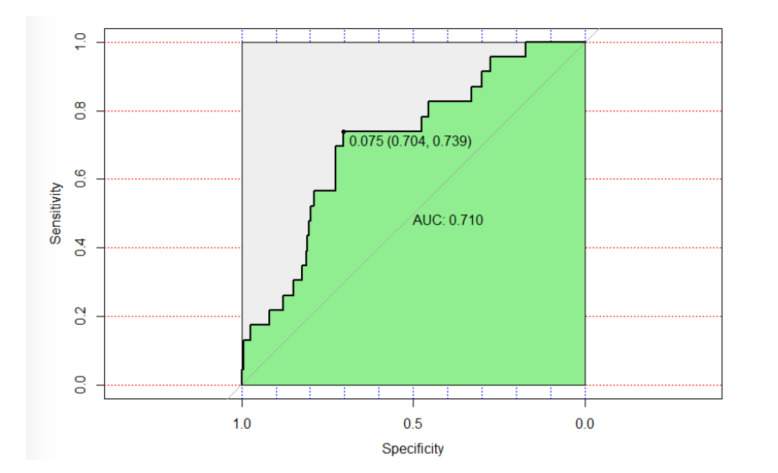
Receiver operating characteristic (ROC) curve.

According to the ROC curve for this model (Figure 2), we have obtained a specificity of 70.4% and a sensitivity of 73.9% for a cohort point of 0.75%. The area under the curve (AUC) was 0.71, indicating a fair discrimination of the model for the predictors of fracture event.

### 3.5. Disease-Free Survival (DFS)

**Figure 3:**
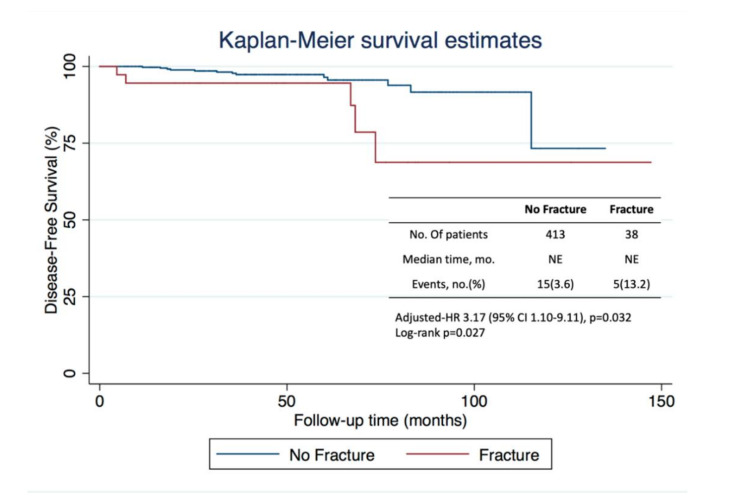
Kaplan–Meier curves for disease-free survival in the analysis population, which included 451 patients. The number of events is the number of events of disease recurrence or death. The median duration of follow-up was 41 months, after which 20 patients had a recurrence, 5 in the fracture group, and 15 in the non-fracture group.

After a median follow-up of 41 months (25.0–62.6), 20 patients (4.4%) had a recurrence: 5 (13.2%) vs. 15 (3.6%) in fracture and non-fracture groups, respectively. As expected, median DFS could not be estimated in neither cohort as it was not yet achieved. Estimated DFS at 73 months was 78.6% (95% CI, 47.67–92.46) in the fracture group and 95.6% (95% CI, 91.25–97.78) (log-rank *p* = 0.027) in the non-fracture group (Figure 3). On multivariate analysis, adjusting for age, staging, and intrinsic BC molecular subtype, an unfavorable prognosis regarding disease-free survival for patients who suffered a fracture event was observed (adjusted-HR of 3.17, 95% CI 1.10–9.11; *p* = 0.032).

## 4. Discussion

With the improvements in the diagnosis and treatment of EBC, and the resulting increase in survival [24], the adverse effects that arise from cancer treatments deserve our full attention and concern. Despite this, the emphasis provided to supportive care is often insufficient and disregards the magnitude of the benefits in terms of quality of life and even survival.

Breast cancer and bone health are two indivisible areas, as the bone is not only the major place for metastasis from this cancer, but it is also tremendously affected by specific cancer treatments. BMD decrease and symptomatic fractures can have important clinical implications, contributing to morbidity and mortality, especially in elderly women, profoundly affecting quality of life, due to incapacity, psychosocial disabilities, and physical limitations [25]. While specific guidelines have been published regarding bone health, real-world data are lacking in what regards adherence to these recommendations and identification of patients at higher risk for skeletal events.

This retrospective study first addressed the potential negative effects that AI, widely used as adjuvant treatment for EBC, can have on bone health. The study cohort included real-world data from 451 women treated with AI with curative intent. The majority of patients had some degree of BMD loss and fracture incidence was in line with previous data reported in the literature [20,23]. Age, as well as time under AI, were relevant independent contributors for fracture incidence, providing important information regarding the deleterious effect of extended therapy with AI on bone health, particularly in older women. Currently, debate still exists on the optimal duration of HT for luminal EBC. Several factors may weigh on this decision, such as disease staging, particularly lymph node involvement; age; and comorbid conditions or risk factors [26,27,28]. Our results suggest that in women with more advanced age, risk factors for recurrence should be well weighted with risk for BMD loss and fracture incidence. Shorter duration of AI therapy using for example a switching strategy with tamoxifen, should be considered. As showed by the MA.17 trial, treatment with tamoxifen prior to AI may counterbalance the deleterious effect of AI in the bone, due to a protective effect on BMD exerted by tamoxifen, which leads to lower fracture incidence [29].

Clinicians adherence to published guidelines was overall low: only 69% of women under AI were receiving concurrent calcium and vitamin D supplementation; baseline bone densitometry was only performed in 7% patients and 31% did not perform this exam during the period with AI treatment. Antiresorptive therapy was prescribed to a minority of women (16%) despite the high prevalence of risk factors for BMD loss and thus with indication for bisphosphonates use.

In our study cohort, fracture event was associated with lower disease-free survival. These data should be interpreted in the appropriate context of a small exploratory retrospective analysis, with important numerical imbalance between the two groups. Nevertheless, our results elicit the impression of a subgroup with more aggressive disease behavior, leading to changes in bone turnover and potentiating fracture risk in addition to the effect produced by AI. An important research led by Kolb et al. already demonstrated the important crosstalk that exists between osteoblasts and breast cancer cells, taking place earlier in the metastatic process [30]. The results of our study help validate this pathophysiologic link between BC and bone metabolism and support the idea of an interaction that begins early in the BC pathway.

The role of bisphosphonates is established in the prevention of TIBL with AI [9,10,13]. Additionally, in postmenopausal women, important results of clinical trials have shown a benefit in survival for the use of bisphosphonates as adjuvant therapy, highlighting the role of these agents in preventing the appearance of metastasis in BC. Indeed, evidence demonstrates their role in decreasing BC recurrence and increasing survival in this group [18,31,32,33]. Therefore, it is currently recommended to administer zoledronic acid, 4 mg by intravenous infusion, every 6 months for 5 years as adjuvant therapy in post-menopausal breast cancer women who are candidates for adjuvant systemic therapy [8,11,34]. With this new evidence for the use of bisphosphonates in EBC, the role of these therapies is being revisited in Oncology Units being expected that bone health will benefit from this.

There are some important limitations to this study. There is a low incidence of the studied endpoints, leading to different distributions of the outcome variables. These differences obviously affect the predictive capacity of the models built and therefore affect further conclusions. Additionally, data on lifestyle factors as diet, exercise, and body mass index are lacking as they were unavailable in medical records. In fact, collection of clinical data from medical records limits the access to certain information and can influence the accuracy of the information. This pertains to the retrospective nature of the study and thus, to all limitations related to retrospective studies. Single-center study is also a limitation.

## 5. Conclusions

Confirmatory data from large prospective cohort studies are required to validate our retrospective results. Despite this, our study challenges some conceptions regarding the attention given to bone health in everyday practice. This is often a forgotten issue with great impact in QoL, and thus it is the clinicians’ responsibility to address bone health regularly and to prevent events that can negatively affect survivorship. Specifically, the early identification of women who may be at higher risk for fracture events and thus are amenable to be considered for shorter duration of AI treatment is warranted.

The poor adherence to existing guidelines is perhaps the biggest barrier to effective approaches to bone health. A better awareness of clinicians regarding currently available recommendations is currently crucial to assure a rigorous and effective translation of the research evidence to the real world. This practice change could have important impact not only in terms of better quality of care but also in reducing health associated costs.

## Figures and Tables

**Table 1:** Demographic and clinical characteristics of the study population.

Characteristics	Whole Population (*n* = 451)	Without Fracture (*n* = 413)	With Fracture (*n* = 38)	*p*-Value
Age (years)	68 (30–98)	68 (30–98)	74 (47–94)	0.006 *
Menopause, n (%)				
Spontaneously	381 (84)	346 (67)	35 (92)	0.175
Ovarian Function Suppression	70 (16)	67 (16)	3 (8)	
Breast Cancer intrinsic subtype				
Luminal A-like	185 (41)	175 (42)	10 (26)	0.054
Luminal B-like	200 (44)	180 (43)	22 (58)	0.157
Luminal B-like, HER2 positive	65 (14)	59 (14)	6 (16)	0.801
Chemotherapy, n (%)	288 (64)	264 (64)	24 (63)	0.637
Radiotherapy, n (%)	353 (79)	327 (80)	26 (68)	0.523
Previous Tamoxifen, n (%)	104 (23)	97 (23)	7 (18)	0.528
Glucocorticoid therapy, n (%)	16 (4)	13 (3)	3 (8)	0.119
Smoking Habits, n (%)	52 (12)	50 (12)	2 (5)	0.220
Alcohol consumption, n (%)	16 (4)	16 (4)	0 (0)	0.223
Previous Fracture, n (%)	33 (7)	28 (7)	5 (13)	0.132
Time under AI	40 (3–114)	38 (3–113)	49 (3–110)	0.027 *
1st bone densitometry, n (%)	311 (69)	284 (69)	27(71)	
Osteoporosis	74 (24)	65 (23)	9 (33)	0.223
Osteopenia	158 (51)	143 (51)	15 (56)	0.605
Normal BMD	79 (25)	76 (27)	3 (11)	0.074
Mean T-score, Lumbar spine	−1.41 (1.36)	−1.43 (1.35)	−1.32(1.46)	0.875
Mean T-score, Femoral	−1.35 (1.11)	−1.32 (1.13)	−1.77 (0.80)	0.066
Fracture, n (%)	38 (8.4)	NA	NA	NA
Calcium/Vitamin D supplementation, n. (%)	305 (69)	281 (69)	24 (67)	0.860
Antiresorptive therapy, n (%)	72 (16)	64 (15)	8 (22)	0.375
Zoledronic Acid	36 (50)	33 (52)	3 (38)	
Alendronate	28 (39)	24 (38)	4 (50)	
Ibandronate	6 (8)	5 (8)	1 (12)	
Risedronate	2 (3)	2 (3)	0 (0)	

Data are median (min–max) or n (%). Abbreviations: BMD, Bone Mineral Density; HER2, human epidermal growth factor receptor 2; NA, not applicable. (* statistically significant value).

**Table 2:** Univariate and Multivariate logistic regression for the outcome fracture. *p*-values were calculated using Wald test (* statistically significant values). All *p*-values < 0.2 in univariate analysis were used in the multivariate model.

Variables	Univariate Analysis			Multivariate Analysis		
	Odds Ratio	95% CI	*p*-Value	Odds Ratio	95% CI	*p*-Value
Age	1.04	1.01–1.19	0.08	1.01	1.01–1.07	0.024 *
Time under AI	1.02	1.01–1.03	0.021 *	1.02	1.00–1.04	0.037 *
Previous ChT	0.08	0.04–1.33	0.63	-	-	-
Previous RT	0.11	0.05–0.21	0.52	-	-	-
Calcium / Vit D suppl.	0.94	0.45–1.92	0.86	-	-	-
Previous Tamoxifen	0.76	0.32–1.78	0.53	-	-	-
Glucocorticoid therapy	2.71	0.74–9.99	0.13	2.88	0.77–10.96	0.11
Smoking Habits	0.42	0.09–1.77	0.24	-	-	-
Alcohol consumption	1	-	-	-	-	-
Previous Fracture	2.15	0.77–5.94	0.14	1.88	0.65–5.47	0.25
Osteoporosis	1.68	0.72–3.93	0.23	-	-	-
Osteopenia	1.23	0.56–2.72	0.61	-	-	-
BMD Loss	1.89	0.68–5.27	0.22	-	-	-

Age was included as continuous variable. AI, Aromatase Inhibitor; Cht, Chemotherapy; RT, Radiotherapy; Vit D, vitamin D; BMD, Bone Mineral Density.

## Data Availability

All data are available upon request to the author.
